# Theory of Mind Deficits and Neurophysiological Operations in Autism Spectrum Disorders: A Review

**DOI:** 10.3390/brainsci10060393

**Published:** 2020-06-20

**Authors:** Maria Andreou, Vasileia Skrimpa

**Affiliations:** Department of English, School of Arts and Humanities, University of Cologne, 50923 Cologne, Germany; vskrimp1@uni-koeln.de

**Keywords:** EEG, autism, theory of mind, adults and adolescents

## Abstract

Theory of Mind (ToM) is a multifaceted skill set which encompasses a variety of cognitive and neurobiological aspects. ToM deficits have long been regarded as one of the most disabling features in individuals with Autism Spectrum Disorder. One of the theories that attempts to account for these impairments is that of “broken mirror neurons”. The aim of this review is to present the most recent available studies with respect to the connection between the function of mirror neurons in individuals with ASD and ToM-reflecting sensorimotor, social and attentional stimuli. The majority of these studies approach the theory of broken mirror neurons critically. Only studies from the last 15 years have been taken into consideration. Findings from electroencephalography (EEG) studies so far indicate that further research is necessary to shed more light on the mechanisms underlying the connection(s) between ToM and neurophysiological operations.

## 1. Introduction

Autism Spectrum Disorder (ASD) is a complex pervasive neurodevelopmental disorder, presenting great heterogeneity with respect to symptomatology and traits. With regards to the degree of severity, ASD reveals impairments in many domains such as social interaction, verbal and non-verbal communication, and restricted and repetitive behaviours [1]. Regarding cognitive and social abilities, it has been shown that individuals with ASD [2] present great variability. The spectrum roughly ranges from high-functioning autism to low-functioning autism associated with learning impairments and disabilities [3].

In most of the cases, ASD is connected with mental disability, difficulties in movement coordination, attention deficits, sleep disturbances and gastrointestinal disorders [4]. However, it is not uncommon for some individuals within the spectrum to achieve high performance skills in visual abilities, music, art and mathematics [5]. Research so far has shown that the appearance of the disorder is estimated at 1–2%, is 4.5 times more frequent in males than in females and could emerge in all national and socio-economic strata [6]. Ιt should be noted that impairment in social skills is one of the fundamental characteristics of the disorder, accompanying the individual throughout his or her lifespan [7].

As advancements in cognitive neurosciences have drawn attention to neurobiological features of ASD, there is a great need to understand the disorder mechanisms. Various theories have been proposed; however, the most prominent to consider for the majority of the social dysfunction traits has been Theory of Mind (ToM), which relates to the ability of individuals to evaluate the behaviour of others on the basis of their own mental states, such as goals, feelings and beliefs [8] and enables the identification of others’ intentions, emotions, as well as self-awareness [9].

## 2. Theory of Mind

ToM is the ability to interpret the mental states of oneself and others [10] and allows individuals to make considerations as well as reasonable explanations regarding the behavioural patterns of others [11]. However, in the case of individuals with ASD, asymmetry between their own knowledge and that of others is often detected [12]. This is why poor performance in ToM tasks is observed in individuals with ASD [13].

Although ToM is unfounded as an exclusive explanation for the characteristics of ASD, the influence of ToM on social skills is paramount [14]. Individuals with ASD show impairments in the reasoning of intentions and emotions that highlight social conventions [15]. The performance of children with ASD in advanced ToM tasks correlates with their social competence; however, the practice of ToM skills in everyday life is often diminished [16]. It is therefore evident that, in spite of the ability of some children with ASD to generate thoughts, beliefs and intentions in ToM tasks, they are unable to implement these skills in social situations [17,18].

Impairments in ToM abilities in children with ASD lead to social, behavioural and communication deficits, as well as discrepancies in social interaction, due to the inability of individuals with ASD to perceive that behaviour is driven by mental states [19,20]. Social dysfunction can be therefore attributed to the delayed or incomplete acquisition of ToM in ASD; however, individuals within the spectrum show individual differences with regards to the acquisition of those skills. In fact, children with ASD who succeed in ToM tasks are considered to be better socially integrated compared to their ASD peers who fail in those tasks [21,22].

Furthermore, factors in ASD such as social and communication experiences, interaction with parents, inability to process information, weak central coherence and lack of motivation, as well as perception problems prohibit the development of ToM [15,22]. Spontaneity in relation to ToM stimuli and reciprocal socio-psychological cues is totally absent in individuals with ASD, even in the case of high-functioning autism. That being said, individuals with ASD exhibit significant deficits in the process of basic emotion recognition judged from information acquired just from the eye gaze of other people. High-functioning individuals within the spectrum are, however, able to interpret mental states based on the whole facial expression [23]. In all cases of ASD adults, though, there is a lack of spontaneous capacity to attribute mental states.

## 3. Mirror Neurons–Mu Suppression

### 3.1. Mirror Neurons and ToM

The “broken mirror neurons” theory has received attention in literature with respect to possible connections between autistic traits and discrepancies in the function of mirror neurons (MN); it is hypothesised to constitute one of the factors responsible for ToM attenuation in individuals with autistic traits, and is linked to the interpretative neurocognitive theory of social and communication impairments in ASD [24].

MN delineate a functional set of neurons located in the cerebral cortex, activated both during the performance of an action, as well as during the observation of the performed action [25]. They were designated as such due to their ability of mirroring behavioural patterns, enabling the observers to encode the intentions behind the observed action sequences and to be in a position to further imitate them [26]. This set of neurons is mainly found in the inferior frontal cortex, the premotor cortex, the supplementary motor area, the primary somatosensory cortex and the inferior parietal cortex [27] and is hypothesised to be directly related to social abilities and skills in primates and humans, including imitation, empathy, ToM and language development [28,29,30]. Due to the fact that individuals with ASD demonstrate impairments in all the aforementioned domains, it is suggested that the system of MN is dysfunctional in ASD [31,32].

The mechanism underlying the activation of MN is strongly linked to imitation ability and imitation-based learning [33], more precisely the imitation of gestural movements and facial expressions [34]. The inferior frontal cortex and the ventral premotor cortex play a compelling role in the action of facial imitation and mimicry, which is essential for empathy to emerge on a neurobiological level [34,35] and mirrors the synchronous coupling of behavioural and emotional development through non-verbal communication [36]. The inferior frontal cortex has a specific significance in the process of defining intentions or goals by delegating those intentions to representations of internal states, as well as to the transmission and perception of emotional states that are connected to the imitation of facial expressions [37].

Imitation processes depend on the perception of action of the sensorimotor system. The prerequisite of these processes is the elicitation of imitation through external motor stimuli that are identified and executed as action that initiates imitation as response to those stimuli [38]. One of the theories that attempt to provide an explanation for the initiation of imitation is the ideomotor theory of action, which suggests that it is not the motor property of action that triggers a reaction, but rather the goal and intention that defines this action. Iacoboni (2009) mentions that “the coactivation of the intended goal and the motor plan required to achieve it—according to the ideomotor framework—is the result of our experience. We have learned the effects of our own actions, and we expect certain effects when we perform certain acts. This previous learning makes it possible that just thinking about the intended goal automatically activates the representation of the action necessary to obtain it.” [39] (p. 655).

MN are theorised to be in the centre of the process of perceiving the intentions behind an act, which further facilitates the emergence and establishment of empathy [40], and plays a significant role for the foundation of common objectives and motives among individuals [41]. Dysfunction in the system of MN in ASD has an impact on the comprehension of action and intention. In particular, individuals with ASD present deficits in perceiving the motor action and the reasoning of an action [42]. It is hypothesised that MN are the substratum of human cognition and social understanding and that their operation facilitates the process of accessing and perceiving the emotional state of others as the result of one being able to reflect one’s own individual internal states and experiences [41,43,44,45,46].

### 3.2. Mu Suppression in Literature

A method to investigate the activation of MN in humans is through measuring *mu* suppression. *Mu* is a type of rhythm that can be described as the frequency band 8–13 Hz and can be detected in an EEG test. The modulations of the power of the *mu* frequency band can provide evidence for the specific functionality of the MN in terms of the comparison between an active condition and a baseline condition [47,48]. It is still under question whether the suppression of the *mu* rhythm is a sufficient method for measuring the operational activation of the Mirror Neuron System (MNS), mostly due to small sample sizes in the research studies (especially when examining atypical populations, such as ASD) or the fact that it is mainly the power modulations of the central electrodes that are taken into consideration [49].

Despite the fact that the theory of broken MN in individuals with ASD has attracted attention, it has also created a debate with regards to its plausibility and application. The hypothesis of dysfunctions in MN accounts for deficits in ToM and imitation, but nevertheless, literature has critically approached the theory, suggesting that the aberrant operation of the system of MN does not provide efficient reasoning for the aforementioned deficits, but that it is sensorimotor impairments in ASD that have an impact on the interpretation of actions. This theory derives from the observation of animal behavioural patterns that indicate the ability of comprehension of action without being in a position to reproduce or imitate it [50]. As a consequence, only a small body of literature has investigated abnormalities in mirror neuron functioning in individuals with ASD, especially in the cases of adolescents and adults. The majority of the studies using brain activity screening techniques focus on the activation of MN in very young populations (children or infants), and their findings demonstrate impairments in the function of these neurons during ToM and imitation tasks [51,52] or gestural movements [53,54]. Although there is an adequate body of literature investigating the involvement of MS in the performance and observation of mentalising tasks in neurotypical adults [55,56], the studies observing this performance in adult individuals with ASD are limited.

Cole et al. [57] examined the activation of MN in young adults with ASD during intention mentalising tasks, in order to detect a possible link between aberrant mirror neuron activity and autistic traits. They recruited 43 participants that matched in terms of age and verbal IQ level, dividing them into three groups according to their level of autistic traits as evaluated by the Autistic Spectrum Quotient (AQ; Baron-Cohen et al., 2001): low AQ (n = 15, mean age = 23.40), high AQ (n = 15, mean age = 24.13) and ASD (n = 13, mean age = 28.30). The participants were required to watch short videos demonstrating motor actions performed by actors that were divided into two categories: a mentalising task and a non-mentalising task. After the end of the videos, they had the task of deciding upon either the intention of the performed action (mentalising) or its success (non-mentalising). The data derived from an EEG screening test performed during the viewing of the tasks, in combination with an eye-tracking test and a Transcranial Magnetic Simulation (TMS)-induced electromyography (EMG). Their EEG findings demonstrated a lower level of *mu* suppression in the right hemisphere in participants within the group with high autistic traits during the mentalising task, which did not, however, correlate with the quality in mentalising performance. On the other hand, a lower performance in the mentalising task positively correlated with a poorer activation of MN in the left hemisphere; nevertheless, this was not linked to the level of autistic traits of the participants. Data derived from TMS revealed no variation between the groups in terms of the activation of MN and its link to performance in mentalising tasks. The hierarchical categorisation of autistic traits was a predictive factor for *mu* suppression in the 8–10 Hz band for the mentalising task and therefore for poorer mirror neuron firing in the right hemisphere. During the non-mentalising task, however, no low level *mu* suppression was detected. The authors attribute the poorer activation of MN in the right hemisphere in individuals with ASD to an abnormal connectivity among MN and the process of mentalising intentions deriving from actions, which is in accordance with the theory of impaired ToM in ASD.

The observation or mentalising of movement, as well as the imitation or execution of the movement, has been associated with the suppression of the *mu* rhythm [58] and has attracted the interest of research, so as to disentangle the relations that underlie the deficits in imitation and reduced *mu* suppression in ASD. In an earlier study, Bernier et al. [59] conducted a study aiming to investigate this link, hypothesising that individuals with ASD will present an impaired imitation ability in correlation with a low suppression of the *mu* wave. They conducted an EEG imitation experiment examining 14 male adults diagnosed with ASD and 15 neurotypical controls, matched in age (18–44 years), gender and intelligence quotient. The experiment included four condition states: (a) resting state, where the participants were required to just sit still, positioning their hands on their lap, (b) observation state, where the participants had to observe a person grasping a manipulandum, (c) execution state, where the participants were instructed to grasp the manipulandum in the exact same way they saw the person doing, and (d) imitation state, where the participants were required to imitate the instructor grasping the manipulandum (experiment adapted from Muthukumaraswamy et al. [60]). The findings with regards to the resting and execution state conditions did not show differences in *mu* suppression between the two groups (reduced in the resting state and increased in the execution state). Nevertheless, the ASD group demonstrated a significantly reduced *mu* suppression in the observation state condition in contrast to the neurotypical controls, which further supported the hypothesis of an impaired execution/observation system in ASD and therefore identified deficits in imitation abilities. The authors, however, observed that this system could not be totally impaired, since individuals with ASD do not entirely lack the ability to imitate but rather demonstrate poor imitation performance. These findings are in alignment with discrepancies in ToM abilities and attenuations in social integration. The study concluded that the execution and imitation of human movement was connected with impairments in *mu* suppression in ASD, further implying a possible involvement of a dysfunctional MS.

Fan et al.’s [61] study dealt with the index of *mu* suppression in relation to the observation/imitation mechanisms, with the intention to challenge the theory of “broken mirror neurons” in ASD. They conducted an EEG study focusing on *mu* suppression as a factor to measure resonance in the sensorimotor system during the observation and imitation of gestural movements. The researchers recruited 20 male adolescents and young adults with ASD (11–26 years) and 20 neurotypical individuals matching the ASD group in terms of age and intellectual abilities. The experiment included an eye-tracking recording and an EEG recording during the conducting of a test containing four conditions: *baseline* condition (observation of a static object on a screen), *hand* condition (observation of a video-recorded gesturing action), *dot* condition (observation of a video with a white dot), *execution* condition (manipulation of an object in the same manner as in the *hand* condition). Their findings constitute strong evidence against the theory of broken mirror neurons in ASD. More in particular, the *mu* suppression occurring from the EEG monitoring under the conditions of observation and imitation of a gestural action did not show significant variation among the two groups. Predominantly, the results did not reveal any correlation between imitation performance and *mu* suppression, contradicting the most up-to-date research findings [32]. The activation of MN in the ASD participants was evaluated as being preserved, and the *mu* rhythm was very close to that of neurotypical individuals, despite the fact that the performance in the imitation condition was significantly lower in the ASD group. The findings also reveal a relation between attenuated communication capacities and a weak *mu* rhythm, indicating a variation in the symptomatology of ASD. Age progression was not found to influence the results in either the ASD or the control group.

When dealing with impaired ToM, empathy is one of the most prominent attenuated social cues, and it is a hallmark of ToM deficits and social discrepancies in ASD. The study of Fan et al. [62] aimed at investigating the empathic and social understanding abilities of neurotypical individuals and individuals with ASD, in order to disentangle the different factors that contribute to the variances with regards to empathic arousal and the perception of social cues in ASD. Their participants consisted of 24 ASD and 21 controls who participated in an fMRI experiment evaluating pain empathy, and 20 adolescents and young adults (16–29 years of age) and 20 age-matched neurotypical controls who participated in an EEG/ERP experiment. A set of 48 images illustrating injured and non-injured body parts were presented, distinguishing between intentional and unintentional injuries as well as individual pain vs. dyad pain situations; these had to be evaluated by the participants with respect to the level of pain. The results of the study demonstrated lower pain thresholds detected in individuals with ASD in comparison to their neurotypical peers, who presented increased hemodynamic responses in SI/SII, stronger N2 but weak responses in the anterior mid-cingulate and anterior insula, and preserved LPP in the view of unintentional body harm, whereas in the case of intentional injuring, they presented reduced LPP and decreased hemodynamic responses in the medial prefrontal cortex. *Mu* suppression and MN activation in view of injuries appeared to be preserved in ASD individuals, similar to the control group, which in combination with an elevated hemodynamic response in the area of the amygdala and higher PPT indicated that individuals with ASD evaluated the pain of others lower due to an impaired perception of social cues. Their emphatic engagement, however, appears to be high, which contradicts the hypothesis of discrepancies in empathy in individuals with ASD.

Another study that examined the hypothesis of attenuated MN activation and *mu* suppression in adults with ASD in terms of decoding the intentions deriving from motion observations and execution is that of Dumas et al. [49]. The aim of the study was to investigate the validity of this hypothesis for the totality of the brain, focusing on two sub-bands of alpha-*mu* bands (8–10 Hz and 10–12/13 Hz), in contrast to other studies that accept a homogeneity of *mu* suppression in terms of frequency (8–13 Hz) and take into account only the electrodes C3/C4, which are located in the centre of the scalp. They examined ten high-functioning adults with ASD and thirty neurotypical individuals matched in terms of age (20–39 years of age) in a three-condition experiment: simple observation of gestures, free imitation of gestures and imitation of a pre-recorded video. Their findings revealed variations in the *mu* response once two bandwidths of alpha-*mu* were considered. More particularly, the differentiation was detected in the upper sub-band of the sensorimotor region in the ASD group under the condition of a gestural observation, whereas the two groups did not show significant variations in the response of the lower sub-band. The increase in the lower *mu* rhythm band was found atypical, whereas in the higher alpha frequency band it appeared to be normal for the observation condition. On the other hand, the responses to the condition of execution were found normal. The study questions the hypothesis of global impairments in the function of MN in ASD, dissociating attenuations in the process of intention perception from them.

As mentioned above, MN are hypothesised to fire during the observation of an act and could possibly be involved in facial-recognition processing as well as mimicry and imitation processes, reflecting emotional states and ToM abilities [63]. Deschrijver et al. [64] questioned the efficacy of dysfunctional MN as the reason behind deficits in motion observation and imitation abilities in individuals with ASD. Their study aimed to shed light on the cognitive processes that underlie imitation control and imitation impairments in ASD, giving emphasis on three EPR components, the P3, the N190 and the RP in terms of congruency in the stimulus conditions. They tested 23 adults diagnosed with High-Functioning Autism and 23 neurotypical controls matched in terms of age (22–46), handedness and gender. The participants were part of an EEG experiment, during which they were required to observe a videotaped hand performing gestures and to execute a pre-instructed hand gesture right after, under three different conditions: a congruent condition, when the action they were required to execute matched the gesture shown in the video, an incongruent condition, when the gesture they were required to execute did not match the gesture shown in the video and a baseline condition, when the hand shown in the video was in a resting state. The study was the first to conduct a neuroimaging experiment examining the imitation–inhibition task. The findings with respect to the P3 ERP component did not confirm the original hypothesis, demonstrating no significant variation between the ASD group and the neurotypical controls. In that respect, both the individuals with ASD and neurotypical participants generated larger numbers of P3 component during the observation of the congruent gestural action with the gesture they intended to perform. The ASD participants showed the ability to distinguish between compatible and incompatible observed gestures to the intended hand gestures when the processing level was higher. With respect to the RP (readiness potential) Laplacian, the findings suggested that the ASD group had a larger number of RP components for the congruent trials than for the baseline trials. The same effect was also observed for the incongruent trials, which elicited larger P3 Laplacian than the baseline trials. That finding suggests that, in individuals with High-Functioning Autism, the cerebral work load in terms of motor preparation is equally high both when observing a compatible or an incompatible gesture to the planned hand gesture, whereas the influence of the baseline condition appears to be neutral. Unexpectedly, no significant variation was indicated among the two groups in terms of compatible and incompatible conditions. The effect of the intended action on the processing of early visual stimuli (N190), as found in previous studies, could not be replicated in this study. The results postulate the theory that automatic imitation does not exclusively depend on the disentangling of socio-cognitive cues but rather on motor preparation, contrasting the hypothesis of dysfunctions in MN in ASD.

The overall results of studies investigating the relationship between *mu* suppression in ASD and dysfunctional MN are far from conclusive. Aberrant *mu* suppression was not found to be systematically associated with dysfunctional MN, which casts doubts on the robustness of *mu* suppression as a reliable proxy for the functioning of MS or/and the appropriateness of the methodological techniques that have been employed so far in relevant research. This calls for a more in-depth examination of the function of MN and impairments of individuals with ASD at the neurobiological level, as well as interventional methods without invasive techniques. An interventional approach that has received attention in research is the Transcranial direct current stimulation (tDCS), which is a non-invasive cerebral stimulation technique that modulates cortical excitability by applying a low direct current through a set of electrodes on the scalp. In recent years, research using tDCS has gained ground as a great opportunity to causally test the role of specific neural circuits in certain motor or cognitive functions [65]. Enhanced cortical excitability is found to be linked to anodal stimulation, whereas a weaker excitability is associated with cathodal stimulation [66]. The technique has already found application in measuring modulations in attenuated mirror neurons aiming at a potential decrease of the clinical manifestations of individuals with ASD [67], and also as a treatment method for other clinical conditions accompanied with cognitive impairments, such as schizophrenia or Alzheimer’s disease [68,69].

Another important point to consider is the connection between ToM, joint attention and brain connectivity. Joint attention in particular is considered to be a predictive marker for ToM, relying on the efficient integration of information regarding mental states of oneself and of others. It therefore requires successful cooperation among the activation of perceptual neural networks. Deficits in joint attention abilities result in impairments in social engagement, constrain shared intent and imitation, and further diminish the chance of social integration and shared experience opportunities [70]. Jaime et al. [71] examined the EEG coherence during the state of perception of compatible and incompatible joint attention as well as an eye-open resting state. The researchers tested 16 high functioning adolescents with ASD (mean age: 16.2 years) and 17 neurotypically developing controls (mean age: 16.5 years). The participants were presented with 12 short video clips containing a moving red dot and a human model, evoking joint attention with two conditions: a congruent one, where the human model was following the dot with their gaze, and an incongruent one, where the human model was not. The findings of the study showed a low alpha coherence in the central-temporal area of the right hemisphere in the ASD group, which is in alignment with the findings of research studies investigating EEG coherence in adults and children [72,73]. The condition of congruence in joint attention perception did not act as an influencing factor for EEG coherence, neither in the ASD group, nor in the control group. The authors interpreted this finding as supporting that adolescents with ASD have no dysfunction in the frontal-parietal attention-oriented network. Overall, the results support the theory of underconnectivity in ASD. The theory of underconnectivity offers a different dimension, postulating that an aberrant frontal-posterior interaction exacerbates the communication and information exchange between the frontal and the posterior regions that are involved in cognitive activities such as joint attention.

Table 1 below provides an overview of the studies that were selected to be reviewed in this paper:.

## 4. Conclusions

ToM is a multifaceted approach, which encompasses a variety of cognitive and neurobiological aspects and has been found to be impaired in individuals with ASD. One of the theories that attempts to account for some of these impairments is that of “broken mirror neurons”, indicating dysfunctions in the proper activation of a neural circuit responsible for the efficient perception of motion activity. The aberrant firing of this neuronal circuit is suggested to have a negative impact on the ability to encode the intentions behind observed actions and further burdens the mechanism that underlies imitation, joint attention, empathy and ToM in ASD. The present review examined the most recent available studies, in particular studies conducted within the past 15 years, with respect to the connection between the function of MN in individuals with ASD and ToM-reflecting sensorimotor, social and attentional stimuli. The neuroimaging studies reviewed in this paper examined the modulation of attenuation of the *mu* rhythm in ASD using EEG screening tests as a marker for measuring MNS activity. The majority of them approached the theory of broken mirrors critically; the results, however, are contradictory, presenting divergent findings in terms of *mu* suppression and its relation to the performance of individuals with ASD in the experimental tasks of these studies. This deviation may be attributed to the large variation of phenotypical symptomatology across the spectrum of autism, as well as to the limitation of the methodological approaches of the research studies, such as limited sample numbers, a restriction to examining only specific cerebral areas, as well as an inadequate connection of the *mu* suppression emergence to other cognitive operations. Nevertheless, the review revealed discrepancies in the function of MNS in ASD, despite the fact that the activity of this neural network is differently interpreted by the researchers of each study. A clear pattern of aberrant *mu* suppression in ASD is, however, indicated in the reviewed studies, without it being exclusively attributed to dysfunctional MN. The role of MN or cerebral motor activation in general has been challenged, even in neurotypically developing infants. The study of Southgate and Begus [74] showed that nine-month-old children demonstrated motor activation in anticipation of an action, regardless of whether the action was within their own skillset of movement or not. More particularly, the study demonstrated independence in terms of coupling the perceived action with a matching motion representation, indicating that the suppression of the alpha wave is linked to action prediction but that it does not necessarily indicate the activation of MN. These findings were interpreted in alignment with the findings of the study of Kilner et al. [75], which demonstrated that MN are involved not only in the observation of an action but also in the anticipation of a motion of another person, which facilitates the prediction of intended action goals before the execution of the action itself. The common outcome deriving from this review is that individuals with ASD exhibit deficits in ToM-related cognitive processes, such as the perception and mentalisation of actions in terms of observation execution and imitation, especially under conditions of unfamiliar social engagement. Impairments in the interpretation of social cues further burden social communication in ASD. It is worth mentioning, however, that the findings of this review suggest a relation between low performance in mentalising tasks, which is nevertheless not correlated to autistic traits. It would therefore be of particular interest to investigate *mu* suppression as a neurophysiological operation and the way in which it is linked to mechanisms such as mentalising. It is crucial to conduct further research, in order to gain a more conclusive insight regarding the mechanisms underlying the connection between ToM and neurophysiological operations.

## Figures and Tables

**Table 1:** Overview of the selected research studies for this review.

Authors	Participants	Method	Findings
[57]	43 (mean age ~25) with autistic traits	EEG, Eye-Tracker, TMS-EMG	Lower level of *mu* suppression in the right hemisphere in ASD during mentalising task. Positive correlation of lower performance in mentalising task with poorer activation of mirror neurons in left hemisphere, but not linked to the level of autistic traits. Autistic traits predictive factor for *mu* suppression in the 8–10 Hz for mentalising task and poorer mirror neuron firing in right hemisphere. During non-mentalising task, no low-level *mu* suppression detected.
[59]	29 (14 ASD and 15 controls) Age 18–44	EEG	Poorer imitation ability in ASD. Significant *mu* suppression in the execution of an action among both groups. In the action observation condition the ASD group showed a reduced *mu* suppression.
[61]	40 (20 male ASD and 20 controls) age 11–26	EEG	No significant variation among groups in *mu* suppression occurring from EEG monitoring of observation and imitation of a gestural action. Stronger *mu* suppression during gestural action observation than dot observation in ASD. No imitation of the observed action while MNS activation intact in ASD. Relation between attenuated communication capacities and reduced *mu* rhythm.
[62]	40 (20 ASD and 20 controls) age 16–29	EEG/ERP, fMRI	Reduced pain thresholds in ASD. Heightened empathic arousal. Attenuated social perception in the view of the pain of others.
[49]	40 (10 ASD and 30 controls) age 20–39	EEG	When *mu* frequency distinguished into two sub-bands, a differentiation observed in the upper sub-band (10–12/13 Hz) of the sensorimotor cortex in ASD in the condition of gestural observation; no significant variation in lower sub-band (8–10 Hz) among the two groups. No globally dysfunctional MNS in ASD.
[64]	46 participants (23 ASD and 23 controls) age 22–46	EEG	No significant variation between ASD and controls in P3 ERP component. Larger number RP (readiness potential) Laplacian both in congruent and incongruent trials in ASD. No effect of intended action on early visual processing detected.
[71]	33 participants 16 ASD (mean age 16.2) and 17 controls (mean age 16.5)	EEG	Low alpha coherence in central-temporal area of right hemisphere in ASD. Condition of congruence in joint attention perception; no influencing factor for EEG coherence in ASD and controls. No dysfunction in frontal-parietal attention-oriented network of adolescent ASD. Support of theory of underconnectivity in ASD.
